# Disphosphonates cardiac uptake in familial amyloid neuropathy: Comparison between DPD and HMDP

**DOI:** 10.1186/1750-1172-10-S1-P41

**Published:** 2015-11-02

**Authors:** Hamza Regaieg, Renata De Paola Chequer, Rana Ben Azzouna, Vincent Algalarrondo, Besma Mahida, Michel Slama, Dominique Le Guludec, François Rouzet

**Affiliations:** 1 Bichat-Claude Bernard university hospital, Department of nuclear medicine, 75018, Paris, France; 2 Antoine Béclère university hospital, Department of cardiology (NNERF), 92140, Clamart, France

## Background

Familial amyloid polyneuropathy (FAP) is a severe hereditary disease, due to production by the liver of a genetic variant transthyretin (TTR) resulting in tissue amyloid deposits. Cardiac involvement is of major prognostic value. Diphosphonate scintigraphy has been proposed as a diagnostic tool for TTR-related cardiac amyloidosis, but there is no consensus on the optimal radiopharmaceutical. Consequently, we compared the cardiac uptake of two 99mTc-labelled tracers: diphosphono-propanedicarboxylic acid (DPD) and hydroxymethylene diphosphonate (HMDP) in patients with TTR-FAP.

## Methods

122 consecutive patients with TTR-FAP were prospectively included and received randomly DPD or HMDP. Acquisitions (whole-body (WB) and chest SPECT) were performed 3 hours after intravenous injection of the tracer. Quantification of myocardial uptake on WB acquisitions was performed by use of the ratio between the geometric mean of either total or average counts of a region of interest (ROI) drawn over the heart area and the WB total or average counts (H/WBtotal or H/WBaverage). Quantification on SPECT acquisitions was performed by the ratio between 3D isocount volume of interest generated over the myocardium and a standard volume in the right lung (H/L). Quantification of soft tissues uptake was performed by use of ratio between average counts of a ROI drawn over the lumbar spine and a ROI drawn over soft tissues of the lower limb (B/ST) on the WB acquisition.

## Results

The DPD and HMDP groups of patients had similar age (62±15 vs. 59±14 years respectively; p=0.3), sex (males: 67% vs. 58% respectively; p=0.4), TTR mutation (Val30Met: 70% vs. 80% respectively; p=0.3) and activity of the tracer (DPD: 713±86 MBq vs. HMDP: 709±124 MBq; p=0.9). Quantitative parameters derived from whole body acquisition were significantly greater with DPD compared to HMDP (H/WBtotal: 3.8±2.7 vs. 2.4±2.1 respectively; p=0.002 and H/WBaverage: 5.2±2.2 vs. 4.3±1.3 respectively; p=0.01) as well as H/L derived from SPECT acquisition (3.9±3.7 vs. 2.0±1.9 respectively; p=0.001). The uptake by soft tissues was also greater in the DPD compared to HMDP group (B/ST: 3.6±2.0 vs. 5.7±2.9 respectively; p<0.0001).

## Conclusion

The present study shows that in patients with TTR FAP, the uptake of DPD in heart and other soft tissues is superior to that of HMDP. This suggests that DPD should be prioritized for initial assessment of patients suspected of cardiac involvement of TTR-related amyloidosis. Further study is required to assess whether this difference impacts the diagnostic performance and whether DPD is more accurate for the assessment of therapy response.

